# Database of recurrent mutations, an unbiased web resource to browse recurrent mutations in cancers

**DOI:** 10.1016/j.isci.2025.114561

**Published:** 2025-12-29

**Authors:** Deepankar Chakroborty, Katri Vaparanta, Bishwa Ghimire, Ilkka Paatero, Kari J. Kurppa, Klaus Elenius

**Affiliations:** 1 Institute of Biomedicine and Medicity Research Laboratories, University of Turku, 20520 Turku, Finland; 2 Turku Bioscience Center, University of Turku and Åbo Akademi University, 20520 Turku, Finland; 3 Turku Doctoral Programme of Molecular Medicine, 20520 Turku, Finland; 4 Research Oncology, Genentech, 1 DNA Way, South San Francisco, CA 94080, USA; 5 InFLAMES Research Flagship Center, University of Turku, 20520 Turku, Finland; 6 Institute for Molecular Medicine Finland (FIMM), Helsinki Institute of Life Science (HiLIFE), University of Helsinki, 00014 Helsinki, Finland; 7 Department of Oncology, Turku University Hospital, 20521 Turku, Finland

**Keywords:** Biocomputational method, Computational bioinformatics, Cancer

## Abstract

Existing cancer-associated variant databases contain biases arising from duplicate entries and the inclusion of targeted sequencing panels, which interfere with accurate estimation somatic mutation frequency in cancer cohorts. To address this, we developed the Database of Recurrent Mutations (DORM), a web resource derived exclusively from whole-genome and whole-exome sequencing data. By filtering out targeted screens and non-recurrent variants, our analysis reveals that mutation recurrence significantly correlates with oncogenic activity, loss of tumor suppressor function, and unfavorable patient prognosis. In a pan-cancer analysis of EGFR, DORM identified frequent mutations outside the kinase domain that are underrepresented in other databases. This resource offers a streamlined, unbiased platform for mutation frequency analysis, enhancing biomarker discovery and the assessment of clinical variant significance.

## Highlights

• DORM is a fast, open-source web resource for analyzing recurrent cancer mutations
• DORM mitigates biases introduced by targeted sequencing and duplicate samples
• Mutation recurrence correlates with oncogenicity and poor patient survival

## Graphical abstract

**Figure:**
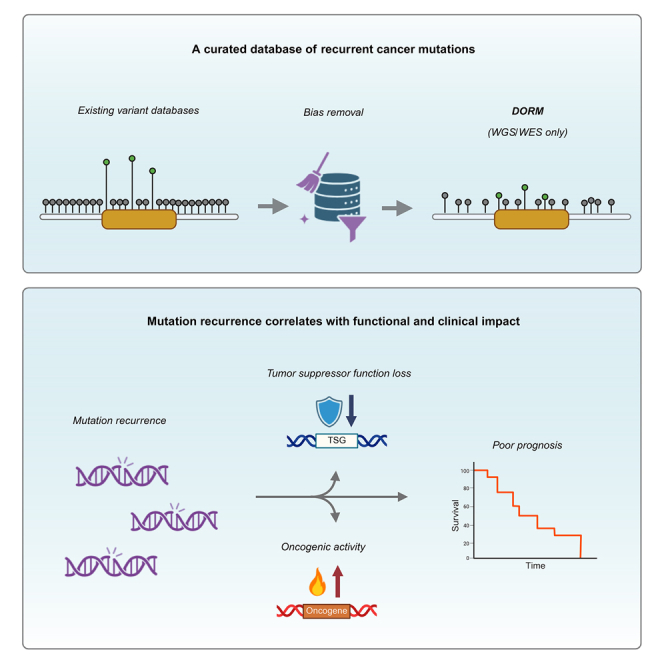

## Introduction

The fast-paced development of next-generation sequencing (NGS) technology and its use to study cancer specimens has led to an accumulation of large quantities of data and the establishment of expansive databases that have propelled the discovery of predictive and therapeutic biomarkers for various cancers.^1^ Large-scale sequencing efforts have pinned somatic mutations as the most common cause of human cancers.^2^ Mutations in several oncogenes are well-characterized driver events in various cancers, e.g., mutations in KRAS G12 residue in pancreatic and lung cancer,^3^ BRAF V600 in melanoma,^4^ and the EGFR L858 in lung cancer.^5^^,^^6^ Despite their frequent observations in the clinic, these hotspot mutations make up only a small proportion of all cancer-associated mutations and there are a large number of recurrent “non-hotspot” mutations.^7^ These recurrent mutations are highly insightful for the underlying biological mechanisms of cancer. Cancer cells are under evolutionary selection pressure and the recurrence of a mutation in the population may indicate its potential to increase cancer cell fitness.^8^

Databases presenting cancer-associated mutations like COSMIC (https://cancer.sanger.ac.uk),^9^ AACR GENIE (https://genie.cbioportal.org),^10^ and cBioPortal (https://www.cbioportal.org/)^11^^,^^12^ present a well-designed interface that provides access to rich data. However, by design, these databases with comprehensive information use a significant amount of bandwidth as well as require multiple steps to access key pieces of information, like the frequency of mutations and the affected amino acid residues. In addition, the mutation frequency information that can be retrieved from these databases without additional manual data processing is affected by several biases. One, the inclusion of information from targeted sequencing can overestimate the mutational frequency of the mutations in the regions included in the targeted sequencing panel design. Consequently, the frequency of the mutations in excluded regions is underestimated due to the increase in the number of samples. Two, the mutation frequency information in the databases can be biased by duplicate records from the same sample. This bias can arise from the inclusion of the same sample in multiple studies as well as the mapping of the mutation to several transcripts. Three, the requirement for the user to select the datasets for the mutation frequency estimation introduces sample-selection bias. Since mutation frequency estimation is continuously used for clinical decision-making as well as biomarker and oncogenic variant discovery, misguided directions in patient care as well as cancer research might be unknowingly selected due to a biased information set.

We sought to address these shortcomings and built a database of recurrent mutations using the large COSMIC cancer registry as a model. Our goal with this project was to develop and deploy a fast and lightweight web-resource to give a user a quick-and-easy way to check the status of a particular mutation of interest in cancer samples in an easy-to-understand format. In addition to direct time-savings, we believe initiatives like ours help further cancer research and its global outreach by improving accessibility to well-summarized and actively debiased information. Moreover, we hope that our open-source framework enables applications to other public cancer registries and diversification to other frontiers of healthcare genomics.

## Results

### Website to browse the recurrent mutations

**Figure 1:**
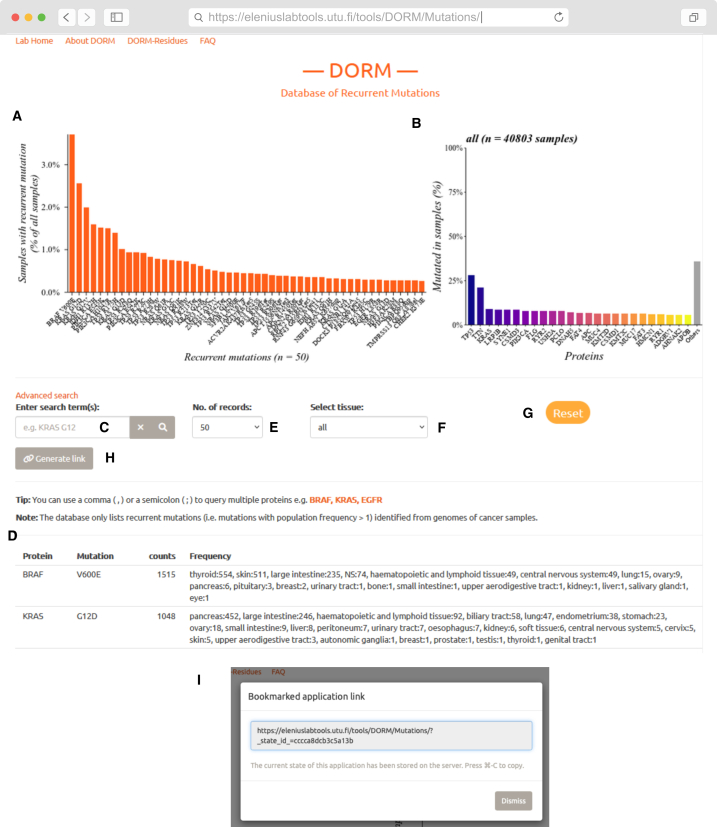
User interface for DORM: Database of recurrent mutations The default GUI of DORM, hosted at https://eleniuslabtools.utu.fi/tools/DORM/Mutations/, shows information about the top 50 most-recurrent mutations identified from genomes of cancer samples. (A) Dynamically updated bar plot that responds to search queries and settings of dropdown menus in “E” and “F.” (B) A bar chart showing the 25 most-frequently mutated genes (color gradient) across all samples in the selected tissue (which can be changed from menu “F”). The “Others” bar represents the percentage of samples not containing mutations in any of the top 25 genes (bars with color gradient). (C) The search bar can be used to query the database with several terms, as well as, regular expressions; an example is displayed in gray text. (D) Table showing the protein name, mutation (displayed as amino acid change), the number of samples with that exact mutation, and the breakdown of the sample count by primary site of the cancer. (E) Dropdown menu that changes the number of records displayed in the table “D” and plotted in the bar plot “A”. (F) Dropdown menu to limit the search to a specific tissue type. (G) Button to reset the website and various parameters to their default values. (H) Button to generate a direct link to repeat a search with the exact search terms and parameters. Clicking this opens the dialogue box “I” which shows the link. (I) Dialogue box showing the direct link which can be used to conduct the exact search again without having to manually enter search term(s) and set the parameters.

The DORM database was created to provide a reliable, fast, protein-centric, and user-friendly resource with reduced bias to analyze substitution mutation frequency in different cancers. The processed database is hosted on a web server at the University of Turku and can be accessed at the URL https://eleniuslabtools.utu.fi/tools/DORM/Mutations (Figure 1). DORM was developed as a tool for cancer researchers with limited bioinformatics expertise. While it can also be used by clinicians, it is intended for research purposes and lacks regulatory approval for diagnostic use. At the top of the page is a plot panel consisting of two dynamic plots that are updated in real-time in response to the user’s search queries. The bar plot on the left shows the cumulative frequency of the individual recurrent mutations in the population (Figure 1A). The bar plot on the right shows the 25 most frequently mutated proteins across all the samples for the selected tissue (Figure 1B). The plot is rendered as a high-resolution image in the user’s web browser following the browser’s dimensions and can be saved as an image directly from the browser. Query term(s) can be entered in the search bar (Figure 1C), which updates the results in the table (Figure 1D) showing the protein, the mutation, the aggregate frequency in the population, and the frequencies categorized by the primary cancer site. The results displayed in the table can be readily copied to a spreadsheet. There is a dropdown menu (Figure 1E) adjacent to the search bar to limit the number of results displayed in the table and the plot. The search or browsing can be restricted to a particular tissue from the menu (Figure 1F). In addition to a button to reset the website and all the parameters to their default value (Figure 1G), there is a button to generate a direct link to a particular search (Figure 1H). Clicking this button opens a dialogue box (shown in Figure 1I), displaying a link that can be used to perform the same search with the exact selected parameters. Clicking this button saves the search term(s) and the set parameter(s) anonymously on our server (i.e., no identifiable information is stored). Such links facilitate the sharing of the results, and make it easy to repeat a search without re-entering the terms and/or setting the search parameters individually. The search bar (shown in Figure 1C) in the DORM database supports advanced search using regular expressions. A brief description and examples of how to query with regular expressions are provided in the supplemental guide in Document S1.

### Mitigating sources of bias in the calculation of mutation frequency

To reduce bias in the mutation frequency estimation, several filtering steps were performed on the source data in COSMIC to create DORM (Figure S1). The source information of all the major cancer databases (like COSMIC, cBioPortal, and GENIE) from multiple institutions share the common problem of mutations being reported multiple times due to the same samples being included in different publications and/or studies. In addition, the source data for the COSMIC database includes individual mutations from the same sample mapped to several transcripts. These duplicate entries constituted a major portion of the source data in COSMIC (71% of all entries) and were consequently removed along with silent mutations and mutations of unknown consequence to create DORM. Since the inclusion of targeted screen data can potentially cause an over-representation or under-representation of genes and their mutations, a phenomenon that has been previously noted,^13^^,^^14^ data from targeted panels and selected sequencing were not included in DORM. Finally, the non-recurrent mutations (61% of non-synonymous coding alterations) were filtered out to minimize the inclusion of mutations derived from sequencing errors.

**Figure 2:**
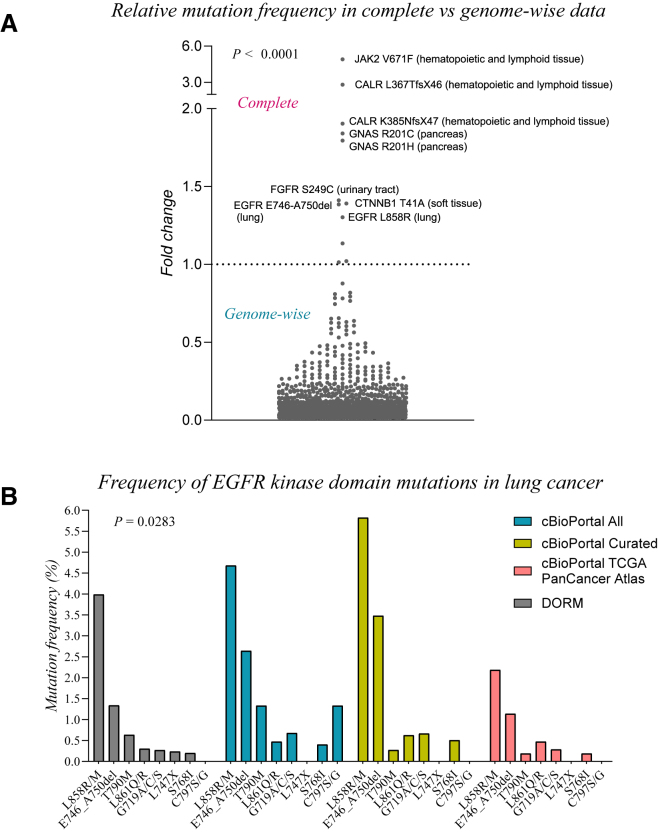
Inclusion of targeted sequencing data and sample selection bias skew mutation frequency estimation (A) Fold change of the relative mutation frequency estimates of somatic mutations in complete (genome-wide and targeted) vs. genome-wide (genome-wide data only) data visualized as a scatterplot. Fold change above 1 or below 1 indicates higher frequency estimates in complete or genome-wide data, respectively. Each dot corresponds to one cancer mutation in a specific tissue. Only mutations with *n* > 10 in the genome-wide data were included in the analysis. (B) Frequency estimates of the EGFR kinase domain mutations in lung cancer. The frequencies were estimated from subsets of studies in cBioPortal or from DORM. All: all lung cancer studies in cBioPortal included for frequency estimation. Curated: lung cancer studies included in the curated set of non-redundant studies (default setting in cBioPortal). TCGA PanCancer Atlas: lung cancer studies included in the TCGA PanCancer Atlas (default setting in cBioPortal).

To confirm that the mutation frequency information in DORM is less biased than in other available resources, the mutation frequency was estimated from the information in DORM and compared to the estimates from other databases. First, the effect of targeted screening data inclusion on the mutation frequency estimation was analyzed. To this end, the mutation frequency was estimated with data from the COSMIC database and DORM. The source data in COSMIC which includes data from targeted screens was processed as the data in DORM. As expected, the frequency of the well-known hotspot mutations such as JAK2 V671F, EGFR L858R, and GNAS R201 C/H was observed to be grossly overestimated when data from targeted screens was included (Figure 2A). In contrast, the mutation frequency of most mutations was underestimated due to the inclusion of the targeted screen data. The mutation frequency of only a few mutations was similar in both cases.

Second, the effect of the bias introduced by sample selection was analyzed by estimating the mutation frequency of the most common *EGFR* kinase domain mutations in lung cancer with cBioPortal and DORM. In the user interface of cBioPortal, the user needs to define the studies to use for the mutation frequency estimation. Two default settings are provided that include either the datasets from TCGA PanCancer Atlas or a curated set of non-redundant studies. The mutation frequency was estimated from the lung cancer datasets from these default selections as well as all available lung cancer study records included in the cBioPortal database (Figure 2B). The different sample selection choices had a significant impact on the mutation frequency estimates (*p* = 0.0238). While the two most frequent mutations were consistently identified across all sample selection choices, the estimates were highly variable even for these recurrent mutations (more than a 2.5-fold difference between the lowest and highest frequency estimates; Figure 2B). These observations indicate that the mutation frequency information is highly influenced by the inclusion of targeted screen data as well as sample selection bias.

### Optimizing the performance of DORM

One of the primary goals of DORM was to display the desired focused statistics and results faster than contemporary databases like COSMIC and cBioPortal. Therefore, we designed DORM as a focused tool and prioritized speed over the breadth of the results. To maximize the efficiency throughout the DORM pipeline, common workflows that can be used to resolve computational bottlenecks were benchmarked (Figure S2). R functions related to reading in data, generating frequency tables, searching, search-replace actions, parallelization, and saving data were benchmarked and selected to optimize the speed of DORM (Figure S2). Google Lighthouse was additionally utilized to benchmark the performance of DORM to understand the end-user’s experience (Figure S3). The Google Lighthouse performance score is the weighted mean of six individual parameters, namely, First Contentful Paint, Speed Index, Largest Contentful Paint, Time to interactive, Total Blocking Time, and Cumulative Layout Shift (details described in the STAR Methods section titled “benchmarking and testing performance”). DORM had a high Lighthouse performance score and behaved well in other Google Lighthouse metrics indicating fast and efficient performance (Figure S3).

### Top recurrent mutations

**Figure 3:**
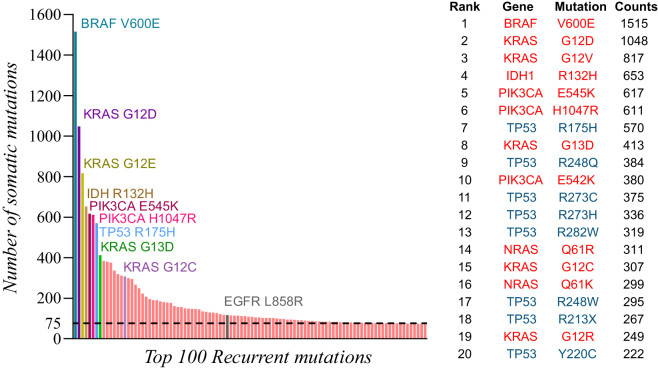
Distribution of the top 100 recurrent mutations Bar plots showing the top 100 most-frequently mutated proteins in the genome-wide somatic mutation data from COSMIC release v100. The top 20 mutations are listed in the table on the right, and the mutations in oncogenes are colored in red and the mutations in tumor suppressors are colored in blue.

The top 100 most frequent mutations across cancer types were assessed with DORM. Among the 100 most-recurrent mutations, the highest number of mutations were reported in TP53 (number of variants [*n*] = 21, frequency in cohort [ν] = 4,346), followed by KRAS (*n* = 9, ν = 3,287), PIK3CA (*n* = 6, ν = 1,907), BRAF (*n* = 1, ν = 1,515), and NRAS (*n* = 6, ν = 1,075) (Figure 2). Among the top 20 recurrent mutations, 12 mutations (ν = 7,220) were in oncogenes, and 8 mutations (ν = 2,546) in tumor suppressor genes. The top three recurrent mutations were the amino acid substitutions BRAF V600E (ν = 1,515), KRAS G12D (ν = 1,048), and KRAS G12V (ν = 817) (Figure 3).

### Mutation recurrence correlates with functional consequence

**Figure 4:**
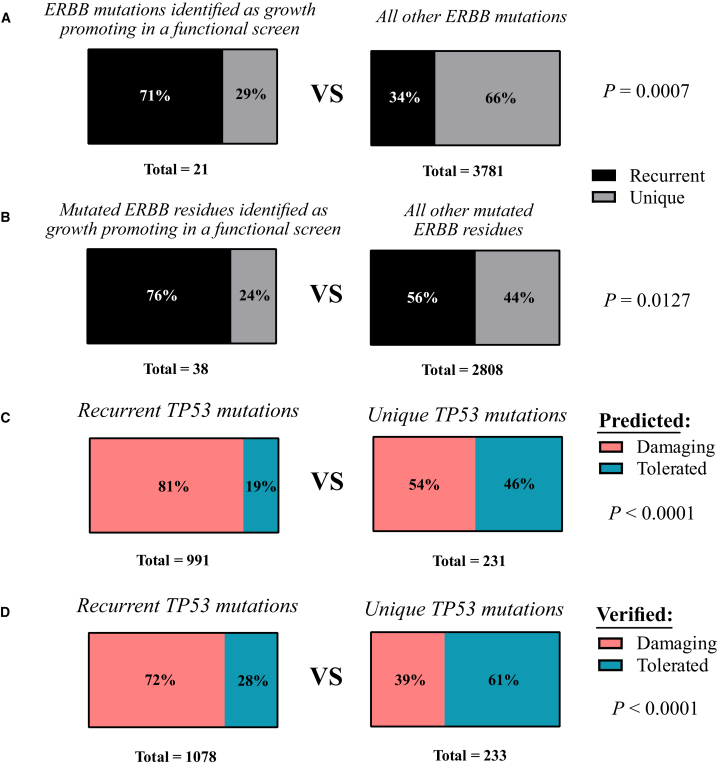
Recurrence is associated with oncogenicity and the loss of function of tumor suppressor genes Recurrent mutations in DORM and unique mutations in COSMIC release v100 were cross-referenced with results from functional screens^18^^,^^19^^,^^20^ and annotations from curated TP53 database.^21^^,^^22^ (A and B) The proportions of recurrent and unique ERBB mutations (A) or mutated residues (B) were compared between those identified in functional screens and those not identified. (C and D) The proportions of predicted (C) or experimentally verified (D) damaging and tolerated TP53 mutations were compared between recurrent and unique TP53 mutations. Fisher’s exact test was used for statistical testing.

We cross-referenced the data from DORM and unique mutations from COSMIC with data from *in vitro* screens of activating mutations (iSCREAM).^15^^,^^16^^,^^17^ Specifically, we compared whether the proportion of recurrent and unique mutations and mutated residues in *EGFR*, *ERBB3*, and *ERBB4* identified in the iSCREAM were different from the proportion of recurrent and unique mutations in all other *EGFR*, *ERBB3*, and *ERBB4* mutations and mutated residues. This comparison indicated that recurrent mutations and mutated residues were enriched in the functional screen, indicating that mutation recurrence is associated with oncogenic properties (Figures 4A and 4B). Several but not all recurrent mutations were observed in these *in vitro* screens. This was not unexpected as it is likely that not all mutations will provide growth advantage in a simplified *in vitro* experiment. Validation of DORM-identified mutations, on the other hand, indicates that at least some recurrent mutations provide a functional growth advantage also *in vitro*, and are more likely to provide it than unique mutations.

As another validation, we compared the recurrent *TP53* mutations in DORM and unique mutations in COSMIC to information in a well-curated *TP53* database.^23^^,^^24^ Indeed, predicted (Figure 4C) and experimentally verified (Figure 4D) loss of function of *TP53* was significantly more associated with recurrent mutations compared to unique mutations. These results indicate that recurrence predicts the functional relevance of a mutation.

### Use case: Pan-cancer analysis of the frequency of EGFR mutations

As a use case, pan-cancer analysis of the frequency of EGFR mutations was conducted with the graphical user interfaces of DORM, COSMIC, and cBioPortal databases. To access the mutation frequency information in COSMIC the name of the gene (EGFR) was entered in the search bar, the correct transcript of the gene was selected, and the information in the “Variants” table of the “Gene” page was manually processed (Figure S4). To access the mutation frequency information in cBioPortal, the datasets used for the frequency estimation were selected, the name of the gene (EGFR) was supplied to the “Enter Genes” box after selecting the “Query by gene” option, and the “Mutations” tab was selected to view the mutation frequency information as a lollipop plot (Figure S5). To access the mutation frequency information in DORM, the name of the gene (EGFR) was supplied to the search bar, and the mutation frequency information was displayed below (Figure S6).

**Figure 5:**
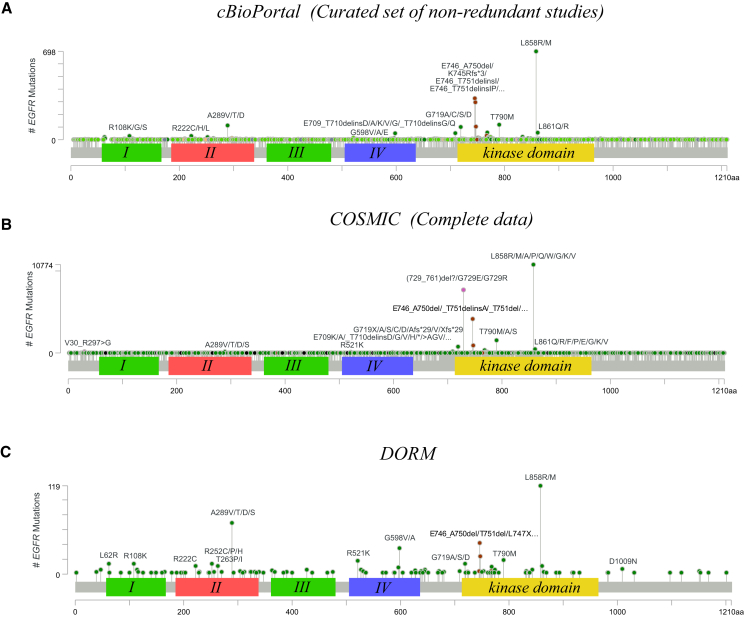
Lollipop plots of the pan-cancer analysis of the frequency of EGFR mutations The frequency of EGFR mutations across cancer types was estimated with the information extracted from cBioPortal (A), COSMIC (B) and DORM (C) databases and visualized as lollipop plots.

The extracted frequency information from the databases was supplied to the MutationMapper tool in cBioPortal to visualize the frequencies as lollipop plots (Figure 5). The L858 alterations were discovered to be the most frequent EGFR mutation in all databases (Figure 5). In both cBioPortal and COSMIC, the frequency of alterations in E746, T790, and G719 in the kinase domain of EGFR (yellow box in Figure 5) were estimated to be more frequent than the mutations in other structural regions. In DORM, however, mutations in other structural regions, such as alterations in residues L62, A289, R521, G598, and D1009 were estimated to be more frequent or similarly frequent as the alterations in E746, T790, and G719. This highlights the markedly different estimates of the frequency of EGFR mutations that can be extracted from DORM and other contemporary databases.

### Mutations in DORM associate with clinical relevance

**Figure 6:**
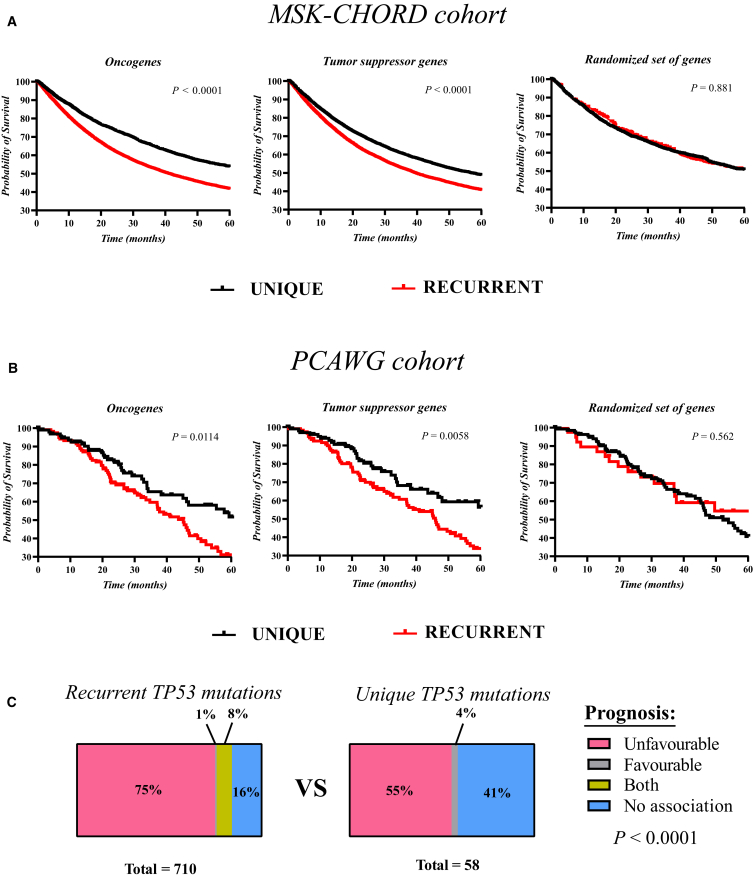
Recurrence of cancer gene mutations is associated with prognostic value (A and B) The overall survival of cancer patients harboring either only unique mutations or at least one recurrent mutation in oncogenes, tumor suppressor genes, or in a randomized set of genes was estimated from the MSK-CHORD cohort^25^ (A) and the PCAWG cohort^26^ (B). The Cancer Gene Census resource^27^ was used as a reference for a list of oncogenes and tumor suppressor genes. The randomized set of genes was generated by random sampling. Mantel-Cox test was used for statistical testing. (C) Recurrent mutations in DORM and unique mutations in COSMIC release v100 were cross-referenced with annotations from the *TP53* database. The proportions of *TP53* mutations associated with either unfavorable (poorer survival, resistance to treatment, or recurrence), favorable or both prognoses as well as non-prognostic mutations were compared between the recurrent and unique mutations. Fisher’s exact test was used for statistical testing.

To assess the clinical utility of the mutations in DORM, we assessed whether recurrent mutations or unique mutations (e.g., the mutations not listed in DORM) would correlate with prognostic value. Existence of a recurrent mutation in at least one oncogene or tumor suppressor gene associated with significantly poorer overall survival compared to the patients who only had unique mutations in oncogenes or tumor suppressor genes in two separate pan-cancer cohorts (Figures 6A and 6B). Recurrent mutations in a randomized set of genes, however, did not have similar effects. This suggests that recurrent mutations in cancer-relevant genes only affect patient survival. To additionally address the potential clinical utility of DORM, we analyzed the relevance of recurrence in the relation of patient prognosis using the information from the curated *TP53* database (Figure 6C). The recurrent *TP53* mutations were significantly more associated with prognostic value than unique mutations, indicating prognostic relevance for mutation recurrence. Taken together, these results indicate that analysis of mutation recurrence provides useful information at the patient level.

## Discussion

NGS of cancer sample series has enabled accurate understanding of cancer biology and helped identify new predictive and therapeutic biomarkers. Here, we present DORM, a fast, less biased and focused (Table 1) web tool, that allows browsing its database derived from an analysis of somatic substitution mutations identified by whole-genome or whole-exome NGS. This strategy avoids the biases introduced due to the use of targeted sequencing panels, inclusion of duplicate entries, and sample selection. Indeed, the mutation frequency estimates of DORM were consistently discovered to differ from estimates derived from other databases that include less filtered data.

**Table 1:** Comparison of search and querying features between DORM and other public databases

Searching and querying	DORM	COSMIC	cBioportal	AACR Genie
Protein	yes	yes	yes	yes
Individual mutations (e.g., KRAS G12C)	yes	yes	yes^a^	yes^a^
Protein sets	yes	no	yes	yes
Tissues	yes	yes	yes	no
Regular expression	yes	no	no	no
Substring search (searching for RAS shows HRAS, KRAS, NRAS, etc.)	yes	yes	no	no

a cBioPortal and Genie require searching for the gene and then the mutation.

DORM only includes recurrent mutations. The biological relevance of recurrent mutations remains to be elucidated and will represent an interesting avenue for scientific research for many years to come. The clearest evidence for relevance is provided by so called “hot-spot” mutations (= highly recurrent mutations), for which there is extensive and long-term evidence for biological significance. For example, KRAS G12^28^ recurrent mutations were identified already in 1980s and have been the subject of extensive research, confirming their functional relevance. Similar evidence for recurrent mutations is available for many other classical oncogenes such as BRAF^29^ and tumor suppressors such as TP53.^30^ In our analyses, mutation recurrence associated with oncogenic properties, loss of function of tumor suppressor genes and prognostic value. Theoretically, these mutations recur because they confer a selective advantage, enhancing the evolutionary fitness of the cancer cells within their specific microenvironment niche.^31^ In addition, some changes may be biochemically more prone to mutation^18^ but without a selection advantage these are less likely to be enriched. In summary, the recurrence of a mutation is an interesting signal and may indicate that the mutation may improve cancer cells fitness. While it is possible that similar mutations occur by chance, the likelihood of that decreases as the frequency of re-occurrence for the specific mutation increases. Frequency estimates may be also confounded by the existence of germ-line mutations. While recurrence of *de novo* germ-line mutations would be of importance, inherited mutations may skew the frequency estimates of somatic mutations. Although the COSMIC database used here accounts for mutation’s somatic status, utilizing additional filters in DORM for germ-line mutations could be useful.

A pan-cancer analysis of *EGFR* mutation frequencies was performed using DORM and other public databases. While frequency estimates from other databases identified the kinase domain of *EGFR* as a hotspot for recurrent mutations, analysis using DORM revealed that additional structural domains also harbor mutations with comparable recurrence rates. If these findings were applied to the design of a targeted sequencing panel, these data would support screening of the entire *EGFR* coding region to capture all frequently occurring oncogenic mutations. In contrast, reliance on other databases would limit screening to the kinase domain, potentially missing patients who could benefit from EGFR-targeted therapies. Indeed, DORM identifies several non-kinase domain *EGFR* mutations in lung cancer that have clinical implications identified in patients. These include three mutations that correlate with response to EGFR-targeted therapy (two in NSCLC and one in glioblastoma),^19^^,^^20^^,^^21^ one associated with anti-EGFR therapy resistance (in colorectal cancer)^22^ and two identified as biomarkers by the FDA.^25^ A classic targeted sequencing screening panel for lung cancer only sequences the *EGFR* exons 18–21 that constitute the kinase domain. This indicates that certain clinically relevant *EGFR* variants are not captured by these targeted sequencing panels. Targeted sequencing panels, however, have been found especially useful in the clinic for assessing tumor mutational burden in low-purity samples.^26^

In addition to enhanced performance and speed DORM offers several advantages (Tables 1 and 2), most notably the ability to directly search for sets of proteins and use regular expressions. Additionally, DORM is the only database that can summarize mutations at the level of amino acid residues (accessible via: https://eleniuslabtools.utu.fi/tools/DORM/Residues/). Data from all other databases require manual processing to retrieve this information. DORM is also the only database that allows the user to view a large amount of data without having to click through numerous pages of results (on DORM, users can choose from a range of 10–10,000 results to display). DORM is also the only database that is free of cookies, trackers, and any embedded analytics.

**Table 2:** Comparison of additional features between DORM and other public databases

Additional features	DORM	COSMIC	cBioPortal	AACR Genie
Direct link to save and share search results	yes	yes	yes	yes
Summarize by residue	yes	no	no^a^	no
Duplicate samples	no	yes	yes	yes
Display most recurrent mutations for a tissue or protein set	yes	no	no	no
Show frequency of a protein being mutated in various tissues	yes	yes^b^	yes	no
Number of rows displayed in table	10–10,000	10–100	25	25
Free from cookies, trackers and/or analytics	yes	no	no	no

a cBioPortal lollipop occasionally groups hotspot mutations at a residue as a single lollipop.

b Possible on COSMIC Cancer Browser.

While COSMIC, cBioPortal and AACR Genie feature duplicate entries, DORM does not. Individual mutations, such as *KRAS* G12C, can be directly searched on DORM as well as COSMIC. On cBioPortal, the implementation of tissue-specific search filters is similar to DORM (i.e., requires selection from a menu), whereas COSMIC requires users to select the tissue from a table in the “tissue distribution” section.

DORM is lightweight, and can be run on standard consumer hardware using our open-source codebase (see STAR Methods for links to the repositories). DORM is publicly available on a virtual private server allowing resources to scale with an increase in demand. We believe that DORM improves the accessibility of important information regarding recurrent mutations by being faster and more resource-efficient than the competition.

While originally designed for cancer researchers, our discussions with oncologists at Turku University Hospital confirmed its potential value in clinical decision-making. DORM’s strengths as a clear, fast, and reliable tool were highlighted particularly in tumor board discussions to identify tumor origin, confirm variant-diagnosis consistency, and assess the functional consequences of rare mutations. Clinicians also noted that reliable updates, error handling, and the inclusion of functional, cancer subtype-specific, and survival data would be essential for broader clinical integration. With these improvements, DORM could complement existing tumor board resources, while in its current form it provides significant value for research, education, and variant annotation in clinical discussions.

### Limitations of the study

In the pursuit of speed and performance, certain trade-offs were made that constitute the limitations of DORM (Table 3). For instance, DORM does not incorporate or display the information about copy number variations or structural variations and excludes all the detailed sample- and study-level information. Instead, DORM is protein-centric and focused specifically on substitutions. Consistent with several other databases in our comparison, DORM does not display gene fusions or non-coding mutations, nor does it allow selecting multiple tissues, or display “lollipop” diagrams which is a visualization tool that places the mutations within the context of the protein’s primary sequence.

**Table 3:** Limitations of DORM in comparison to other public databases presenting somatic mutations identified from cancer samples

Limitations of DORM	DORM	COSMIC	cBioportal	AACR Genie
Copy number variations and structural variations	no	yes	yes	no
Show Lollipop diagram for locating mutations on peptide	no^a^	no	yes	no
Show detailed information (sample and study level)	no	yes	yes	yes
Non-coding mutations	no	yes	yo	no
Fusions	no	yes	yes	yes
Select multiple tissues	no	yes^b^	yes	no
Data download	no	yes	yes	no

a DORM shows the distribution of mutations in a single protein in different tissues with a pie chart.

b Possible on COSMIC Cancer Browser.

Tumor heterogeneity, variability in tumor purity,^27^ and the presence of subclonal mutations can lead to underestimation of mutation frequencies, typically in samples with low relative cancer cell content. This inherent bias may affect the frequency estimates in DORM. However, since tumors are always composed of diverse cell types in addition to malignant cells, this issue is not merely an artifact but an informative biological feature of tumors.^32^ Both tumor composition/purity and subclonal mutations are important topics and could be best addressed using single-cell genomics.^33^ This represents a promising avenue for future research as single-cell data are accumulating. The frequency estimates in DORM may be refined as single-cell genomic data achieve sufficient sequencing depth and scale.

As the absolute “ground-truth” of the mutations observed in these large-scale sequencing datasets is unknown, the exact rate of bioinformatic artifacts cannot be estimated. Such artifacts can arise from data processing of including raw data, base calling, quality filtering, and database transfer.^34^^,^^35^^,^^36^ Since all modern sequencing data are subject to numerous bioinformatic procedures and may therefore contain bioinformatics artifacts, reaching absolute certainty regarding the absence of artifacts is theoretically and epistemologically impossible. Consequently, it is not possible to fully ascertain that all mutation counts in DORM (reflecting the number of times a variant is observed) represent true positives rather than bioinformatic artifacts. However, because DORM lists only recurrent mutations, the probability that a specific mutation entry within the database is a false positive due to recurring data artifacts in the exact same genetic region, remains infinitesimally small.

## Resource availability

### Lead contact

Requests for further information and data should be directed to and will be fulfilled by the lead contact, Professor Klaus Elenius (klaus.elenius@utu.fi).

### Materials availability

This study did not generate any new materials.

### Data and code availability

The code for creating and validating DORM has been deposited to GitHub and is publicly available through the following GitHub Repository URLs: https://github.com/KE-group/DORM-2022; https://github.com/dchakro/DORM_Mutations; https://github.com/dchakro/DORM_Residues; https://gist.githubusercontent.com/dchakro/8b1e97ba68563dd0bb5b7be2317692/raw/parallelRDS.R. The source data utilized to create DORM is available on the COSMIC^9^ downloads page (https://cancer.sanger.ac.uk/cosmic/download/cosmic) and NCBI’s RefSeq database^37^ (https://www.ncbi.nlm.nih.gov/refseq/MANE/).

## Acknowledgments

The Cancer Foundation Finland, Novo Nordisk Foundation, Research Council of Finland, Sigrid Juselius Foundation, and Turku University Central Hospital are acknowledged for financial support. The authors wish to thank oncologists Dr. Erika Alanne and Dr. Maria Sundvall (both from Turku University Central Hospital, Finland) for valuable comments on the clinical applicability of DORM.

## Author contributions

Conceptualization, D.C., K.E., I.P., and K.J.K.; methodology, D.C. and K.V.; formal analysis, D.C. and K.V.; funding acquisition, K.E.; investigation, D.C. and K.V.; software, D.C., K.V., and B.G.; data curation, D.C. and K.V.; supervision, K.J.K. and K.E.; visualization, D.C. and K.V.; writing – original draft, D.C., K.V., and K.E.; writing – review and editing, D.C., K.V., K.E., and I.P.

## Declaration of interests

K.E. declares research agreements with Boehringer Ingelheim and Puma Biotechnology and ownership in Abomics, Novo Nordisk, Orion, Roche, and Vertex Pharmaceuticals outside of the submitted work. D.C. declares current employment with Roche and Genentech.

## Declaration of generative AI and AI-assisted technologies in the writing process

Portions of the article text were edited with the assistance of OpenAI’s ChatGPT-4 and ChatGPT-5 to improve clarity. The authors reviewed and revised all generated content as needed and take full responsibility for the final text.

## STAR★Methods

### Key resources table

**Table:** 

REAGENT or RESOURCE	SOURCE	IDENTIFIER
**Deposited data**		

COSMIC v100 Genome Screen Mutants data	COSMIC^9^	https://cancer.sanger.ac.uk/cosmic/download/cosmic/v100/genomescreensmutanttsv
COSMIC v100 Samples data	COSMIC^9^	https://cancer.sanger.ac.uk/cosmic/download/cosmic/v100/sample
MANE Select data	RefSeq^38^	https://www.ncbi.nlm.nih.gov/refseq/MANE/
Tumor variants in human tumor samples data file	TP53 database^23^	https://tp.cancer.gov/
Prognostic value of tumor variants file	TP53 database^23^	https://tp.cancer.gov/
Cancer Gene Census data	COSMIC^9^	https://cancer.sanger.ac.uk/census
MSK-CHORD cohort data	cBioPortal^12^	https://cbioportal-datahub.s3.amazonaws.com/msk_chord_2024.tar.gz
PCAWG cohort data	cBioPortal^12^	https://cbioportal-datahub.s3.amazonaws.com/pancan_pcawg_2020.tar.gz

**Software and algorithms**		

Prism v9 and v10	GraphPad	https://www.graphpad.com/
R	The R project^39^	https://www.r-project.org/
HTML5	WHATWG	https://html.spec.whatwg.org/multipage/
CSS	WORLD WIDE WEB CONSORTIUM, Cascading Style Sheets (CSS) Working Group	https://www.w3.org/Style/CSS/Overview.en.html
JavaScript	MDN	https://developer.mozilla.org/en-US/docs/Web/JavaScript/Reference
AWK	GNU^40^	https://www.gnu.org/software/gawk/
R shiny	Posit^41^	https://shiny.posit.co/
GNU Gzip	The Free Software Foundation	https://www.gnu.org/software/gzip/
R data.table package	Barrett et al.^42^	https://r-datatable.com/
R stringi package	Gagolewski, M.^43^	https://stringi.gagolewski.com/index.html
R jsonlite package	Ooms et al.^44^	https://cran.r-project.org/web/packages/jsonlite/index.html
R microbenchmark package	Mersmann et al.^45^	https://cran.r-project.org/web/packages/microbenchmark/index.html
Pigz	Adler, Mark.	https://zlib.net/pigz/
NGINX	NGINX	https://nginx.org/
Transport Layer Security (TLS) 1.3	Rescorla, E.^46^	https://www.rfc-editor.org/rfc/rfc8446.html
Advanced Encryption Standard (AES-256)	National Institute of Standards and Technology	https://doi.org/10.6028/NIST.FIPS.197
Google Lighthouse	Google	https://developer.chrome.com/docs/lighthouse/overview
MutationMapper	cBioPortal^12^	https://www.cbioportal.org/mutation_mapper

**Other**		

Resource website for the DORM database	This paper	https://eleniuslabtools.utu.fi/main/docs/DORM.html

### Method details

#### Website and web server

The DORM database is accessible at https://eleniuslabtools.utu.fi/tools/DORM/Mutations/, and all requests to the server are handled by an NGINX reverse-proxy (https://nginx.org/) that encrypts the traffic between our server and the end-user’s web-browser. The connection is encrypted using the latest Transport Layer Security (TLS) cryptographic protocol 1.3^38^ and an industry standard 256-bit Advanced Encryption Standard (AES-256).^39^ As a fallback, the server of DORM also supports connections over TLS 1.2 to support legacy hardware and browsers. The landing page website and the documentation is built using HTML5, CSS and JavaScript. The web tools are built using Shiny^40^ and R.^41^ These services are hosted on a virtual private server at the premises of University of Turku, Turku, Finland. The source code for deploying DORM as an R Shiny app is available at https://github.com/dchakro/DORM_Mutations and https://github.com/dchakro/DORM_Residues repositories.

#### Hardware

*Database processing & analysis*: Apple iMac (early 2013) equipped with Intel Core i5 CPU (4 cores – 3.2 GHz), 24 GB DDR3 RAM, 500 GB SSD running macOS Catalina 10.15.

*Server*: Virtual private server (KVM virtualization) with Intel(R) Xeon(R) Gold 5120 CPU (1 core – 2.20 GHz), 6 GB ECC RAM, 100 GB HDD running Ubuntu 22.04 LTS.

*Web performance testing*: Apple MacBook Pro (early 2015) equipped with an Intel Core i5 CPU (2 cores – 2.7 GHz), 8 GB DDR3 RAM, 500 GB SSD running macOS Catalina 10.15. The device was connected via a 5 GHz Wi-Fi router to the public ISP (i.e., outside the network where the DORM database is hosted) over a 100 Mbps fiber optic broadband connection.

#### Data and processing of data

Data were acquired from COSMIC release v103 (released November 18, 2025 https://cancer.sanger.ac.uk) as a GNU zip (GZIP) archive of the tab-delimited text file with all mutations identified from genome-wide screens (includes data from whole genome sequencing, and whole exome sequencing). The samples from targeted screens were excluded to ensure our analysis is free from selection bias and to facilitate the direct comparison of the frequency of mutations between different proteins in a particular tissue. No additional germline filtering was performed. As a result, DORM includes: (i) recurrent mutations reported as somatic in other cancer samples, (ii) recurrent mutations confirmed as somatic by comparison of tumor and normal tissue, and (iii) recurrent mutations with unconfirmed somatic status due to lack of normal tissue sampling. As COSMIC does not report metrics for tumor purity or subclonality, these characteristics are unknown for the recurrent mutations in DORM. No additional orthogonal validation was performed.

*Pre-processing*: The decompressed data is processed using the “awk” programming language^42^ to select relevant columns (named, Gene name, Sample name, Primary site, Primary histology, Genome-wide screen, Mutation CDS, Mutation AA). The selected columns were read in R by using the data.table::fread() function.^43^ The complete database was stored as standard R object in the .RDS file format, with a notable difference: instead of saveRDS from R base, which uses serialized compression, parallelized GZIP (pigz: https://zlib.net/pigz/) was used for compression – decompression. The functions for reading-writing R objects in .RDS files using parallelized compression-decompression are described in this R script.

*Filtering*: The duplicate entries for mutations mapped to other than the MANE select transcripts^44^ were removed (Figure S1). Mutations with unknown consequences on the protein level were removed. From these, silent mutations were removed (Figure S1). To prevent redundant counting of the same mutation, a unique identifier was generated for each mutation using the sample name, protein name, and amino acid change. Entries with identical mutation IDs were considered duplicates and removed from the dataset (Figure S1). Mutations with single occurrences (i.e., frequency = 1) were removed from the list of unique coding mutations, as they are not part of the pool of recurrent mutations. The filtered database with unique coding mutations was stored as a parallelized GZIP .RDS file, enabling faster load times. Searching and parsing of the text was performed with the ‘stringi’ R package.^45^

*Processing*: For each mutation, its cumulative frequency of occurrence, as well as its frequency in cancers of various tissues, was calculated and compiled into a table. The table was sorted by mutation frequency (total number of samples across all cancers) and then stored as a parallelized-GZIP .RDS file.

*Updates*: Since 2004, marking the release of COSMIC v1, the dataset has been updated on average four times per year (range: 11 releases in 2006 and two releases in 2020). The COSMIC data releases need to be acquired from (https://cancer.sanger.ac.uk), then our optimized pipeline can be run with a shell script that automates the processing and generation of the underlying database for DORM.

#### Benchmarking and testing performance

To evaluate the performance of different code blocks, the ‘microbenchmark’ R package^46^ was used to gather data. The data were graphically represented using Graphpad Prism 9 and 10.The code blocks used for testing and benchmarking their performance is available at https://github.com/KE-group/DORM-2022 repository.

The performance of the websites hosting the databases was measured on Google Chrome (v. 97.0.4692.99) with Google Lighthouse (v. 8.5.0) (available in Chrome DevTools). Lighthouse (https://github.com/GoogleChrome/lighthouse) is an open-source tool for automated auditing and assessing performance metrics. A search for EGFR mutations was performed on the five databases (DORM, COSMIC, ICGC,^47^ cBioPortal and AACR GENIE), and links (Table S1) to those individual searches were used to test the performance of the databases. This was performed to discount the varying duration required to do the same search on the four databases. Lighthouse 8 produces a performance score which is a weighted average of First Contentful Paint (10%, marks the time at which the first text or image is painted), Speed Index (10%, shows how quickly the contents of a page are visibly populated), Largest Contentful Paint (25%, marks the time at which the largest text or image is painted), Time to interactive (10%, the amount of time it takes for the page to become fully interactive), Total Blocking Time (30%, measures the total amount of time that a page is blocked from responding to user input), and Cumulative Layout Shift (15%, measures the unexpected movement of page content). The JSON data in the lighthouse reports was parsed using the ‘jsonlite’ R package^48^ and tabulated in R. The data were graphically represented using GraphPad Prism 9.

#### Mutation frequency estimation

The frequency of a mutation was estimated by dividing the number of patient samples with the mutation by the total number of patient samples from the same cancer type. To analyze the effect of targeted data inclusion on the mutation frequency estimation, the “Genome Screens Mutants” and “Targeted Screens Mutants” data in the download page of the COSMIC database v100^9^ were processed as the data in DORM.

To analyze the effect of sample selection bias on mutation frequency estimation, the frequency estimates for common EGFR kinase domain mutations were acquired from cBioPortal v 6.0.12^12^ and DORM. Either all lung cancer studies, lung cancer studies related to the TCGA PanCancer Atlas or lung cancer studies included in the curated set of non-redundant studies were selected for the frequency estimation.

To perform the pan-cancer analysis on the frequency of EGFR mutations, frequency estimates from DORM, COSMIC, and cBioPortal databases were acquired. The mutation frequency information in COSMIC was acquired from the “Variants” table of the EGFR Gene page in COSMIC. The studies included in the “curated set of non-redundant studies” default setting were used for the frequency estimation in the cBioPortal database. The lollipop plots were drawn with the MutationMapper tool in cBioPortal.^12^

#### Comparative evaluation of recurrent and unique mutation effects

Recurrent mutations from DORM and unique mutations from COSMIC v100, which included all mutations in COSMIC that are not listed in DORM, were used for the analyses. The ERBB mutations identified as growth promoting were sourced from the results of *in vitro* screen of activating mutations.^15^^,^^16^^,^^17^ The predicted and experimentally verified functional classifications of *TP53* mutations were sourced from the *TP53* database.^23^^,^^24^ Variants classified as damaging or partially damaging in at least two out of five prediction methods in the database (BayesDel, REVEL, AGVGDClass, SIFTClass, Polyphen2) were considered as predicted damaging. Variants classified as damaging or partially damaging in the results of at least one out of three functional screens in the database (TransactivationClass, DNE_LOFclass, DNEclass) were considered as verified damaging.

The prognostic value of *TP53* mutations were sourced from the *TP53* database. Mutations included in studies where *TP53* mutations were associated either with treatment resistance, poorer survival or tumor recurrence were considered unfavourable prognoses. In contrast, *TP53* mutations included in studies where *TP53* mutations were associated with survival, treatment response or no relapse were considered favourable prognoses. *TP53* mutations that were associated with unfavourable and favourable prognoses in separate studies were categorized as both. The mutations that had no prognostic association in the studies were categorized as no association.

Survival analysis of patients in the pan-cancer MSK-CHORD^49^ and PCAWG^50^ cohorts were conducted using the data sourced from the cBioPortal database.^12^ The overall survival was analyzed. All patients with at least one recurrent mutation in any oncogene, tumor suppressor gene or a randomized set of genes were assigned to the recurrent group. Patients with only unique mutations in oncogenes, tumor suppressor genes or a randomized set of genes were assigned to the unique group. The list of oncogenes and tumor suppressor genes were sourced from the Cancer Gene Census resource.^1^ The randomized set of genes was generated by random sampling of all mutated genes. The size of the randomized sets was set to equal the number of oncogenes and tumor suppressor genes in the Cancer Gene Census resource (n=325). The process was repeated 10 times and a representative case was visualized.

### Quantification and statistical analysis

Statistical testing comparing multiple groups in testing code block performance was performed using Brown Forsythe and Welch ANOVA test and correction for multiple testing was done by controlling the false discovery rate using the two-stage step-up method of Benjamini, Krieger and Yekutieli^51^^,^^52^ in Grahpad Prism 9. Statistical testing comparing two groups of observations was done using Welch’s t-test in Grahpad Prism 9. The statistical significance of the relative fold change of the relative mutation frequency in complete (genome-wide and targeted) vs genome-wide data was calculated with one sample Wilcoxon test against the hypothetical median value of 1. The statistical significance of the sample selection to the mutation frequency estimates was calculated with a two-way ANOVA. The normality and homoscedasticity assumptions were tested with D'Agostino-Pearson omnibus, Anderson-Darling, Shapiro-Wilk, Kolmogorov-Smirnov, and Spearman’s tests. The statistical significance of the proportions of unique and recurring mutations and mutated residues in the groups of ERBB mutations identified in the screens and all other ERBB mutations, and damaging and tolerated mutations in the groups of recurrent and unique *TP53* mutations was estimated with the Fisher’s exact test. The statistical significance of the proportions of mutations associated with unfavourable, favourable and both prognoses as well as mutations with no prognostic value in the groups of recurrent and unique mutations was estimated with the Fisher’s exact test. The statistical significance of the difference in survival of the recurrent and unique mutation groups was estimated with the Mantel-Cox test. Statistical parameters such as what individual points represent are reported in the Figure Legends.

### Additional resources

Access to the DORM database and its documentation and FAQs is available through the weblinks: https://eleniuslabtools.utu.fi/main/docs/DORM.html; https://eleniuslabtools.utu.fi/main/docs/DORM-FAQ.html.
