# Supplementary material for: Database of recurrent mutations, an unbiased web resource to browse recurrent mutations in cancers
Source: iScience. 2025 Dec 29;29(2):114561. doi: 10.1016/j.isci.2025.114561 (PMC12818146; doi:10.1016/j.isci.2025.114561)
Supplement: Document S1. Figures S1–S6 and Table S1 [file mmc1.pdf]

iScience, Volume *29*

Supplemental information

Database of recurrent mutations, an unbiased web resource to browse recurrent mutations in cancers

Deepankar Chakroborty, Katri Vaparanta, Bishwa Ghimire, Ilkka Paatero, Kari J. Kurppa, and Klaus Elenius

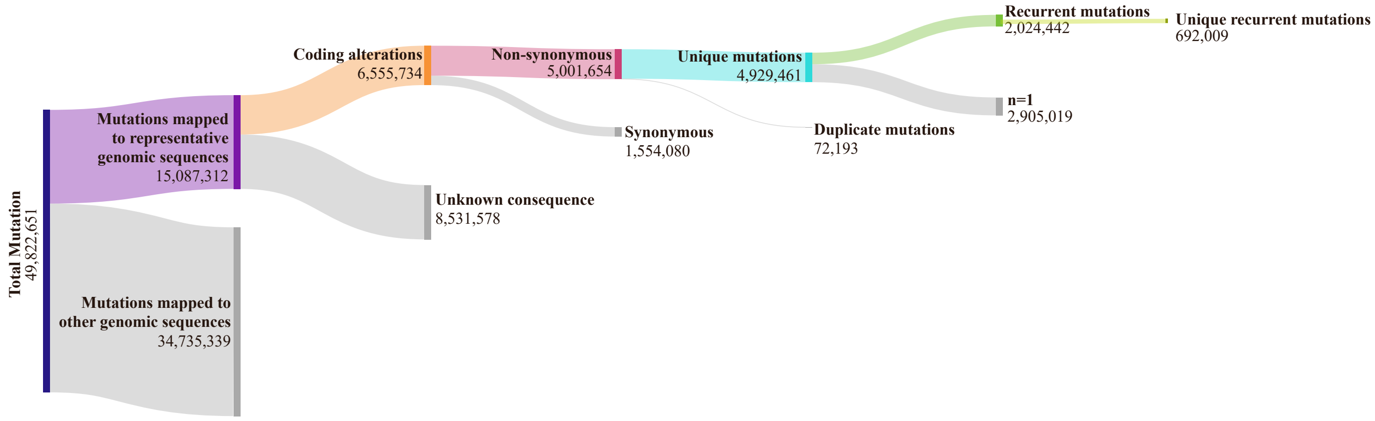

Figure S1. Filtering scheme to isolate recurrent mutations from COSMIC data.
Out of 49.8 million mutations, 34.7 million mutations were removed as they can be attributed to duplicate transcripts of genes. 8.53 million mutations with unreported / unknown consequence were removed. 1.55 million silent mutations were removed, which do not produce any change in the protein. Subsequently, 72,193 duplicate records were removed as they are present due to incorporation of some samples in multiple studies. After this filtering process, 4.92 million unique coding mutations that remained were processed to create the DORM database which summarizes the 2.0 million recurrent mutations (mutations with tissue-agnostic population frequency >1). These 2.0 million recurrent mutations constitute from 0.69 million unique mutations.

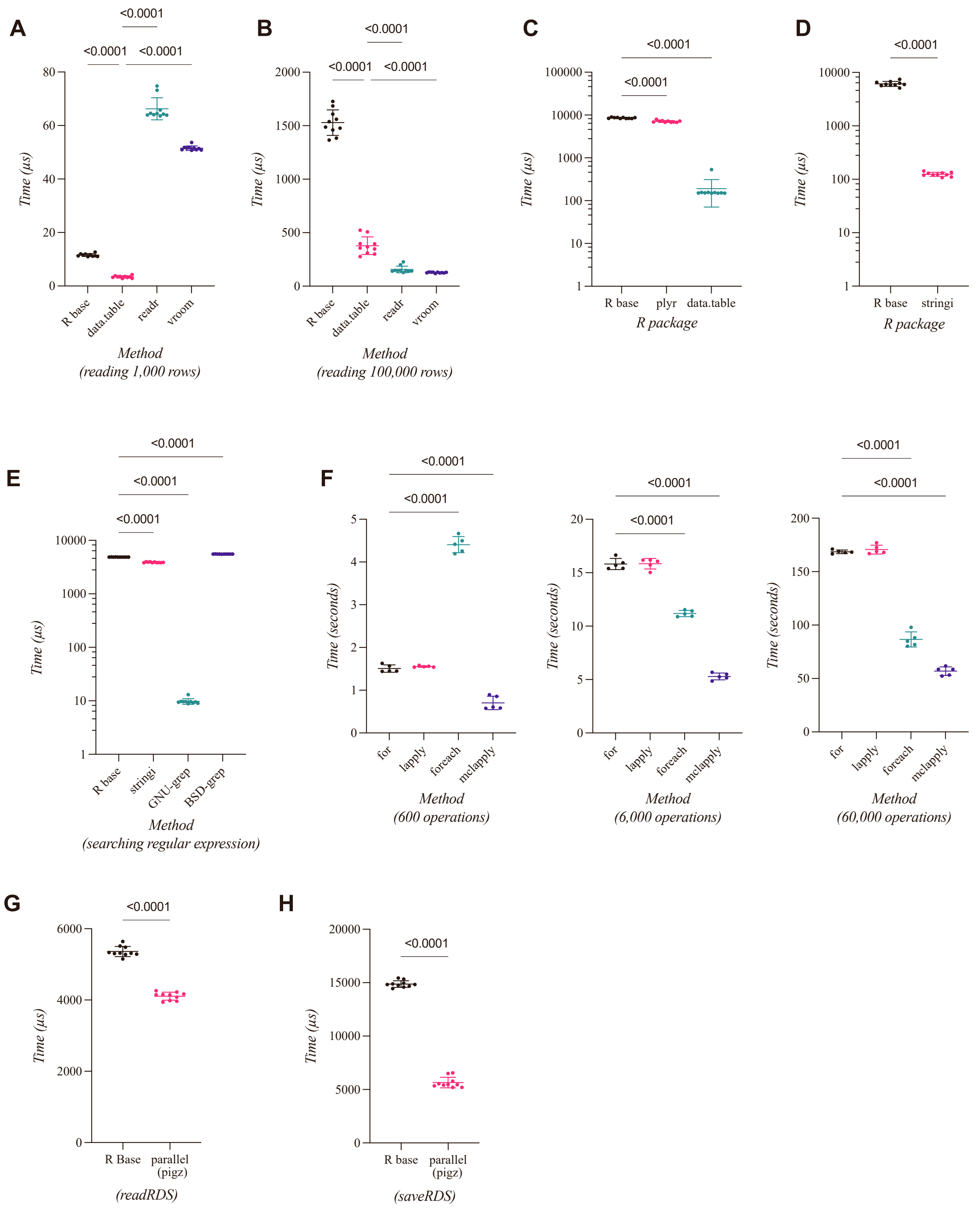

Figure S2. Comparison of strategies and methods for performing various computational operations. Scatter plots showing mean and standard deviation of computational time of different functions. A-B) Different R packages for reading a tab-separated value (TSV) file or 1000 rows (shown in A) and 100,000 rows (shown in B). C) Various approaches of generating a frequency table. D) Searching for a regular expression (pattern: [ACDEFGHIKLMNPQRSTVWYX]?[0-9]+) using R base and stringi package. E) Searching the data with a regular expression (pattern: EGFR|ERBB[2-4]|[HKN]RAS\>) using the indicated methods. For giving the functions in R the best chance, the data was preloaded in the R outside the code for timing the benchmark. F) Comparing various looping constructs for 600, 6000 and 60000 operations. For and lapply are serialized loops, foreach and mclapply are their parallel alternatives. G) Writing an R object containing a table (1 million rows) as an RDS file with either the R base serialized version or our custom parallelized version using 'pigz' for compression. H) Reading an RDS file (written with our parallel version of saveRDS) with the R base serialized version or our custom parallelized version using 'pigz' for decompression.

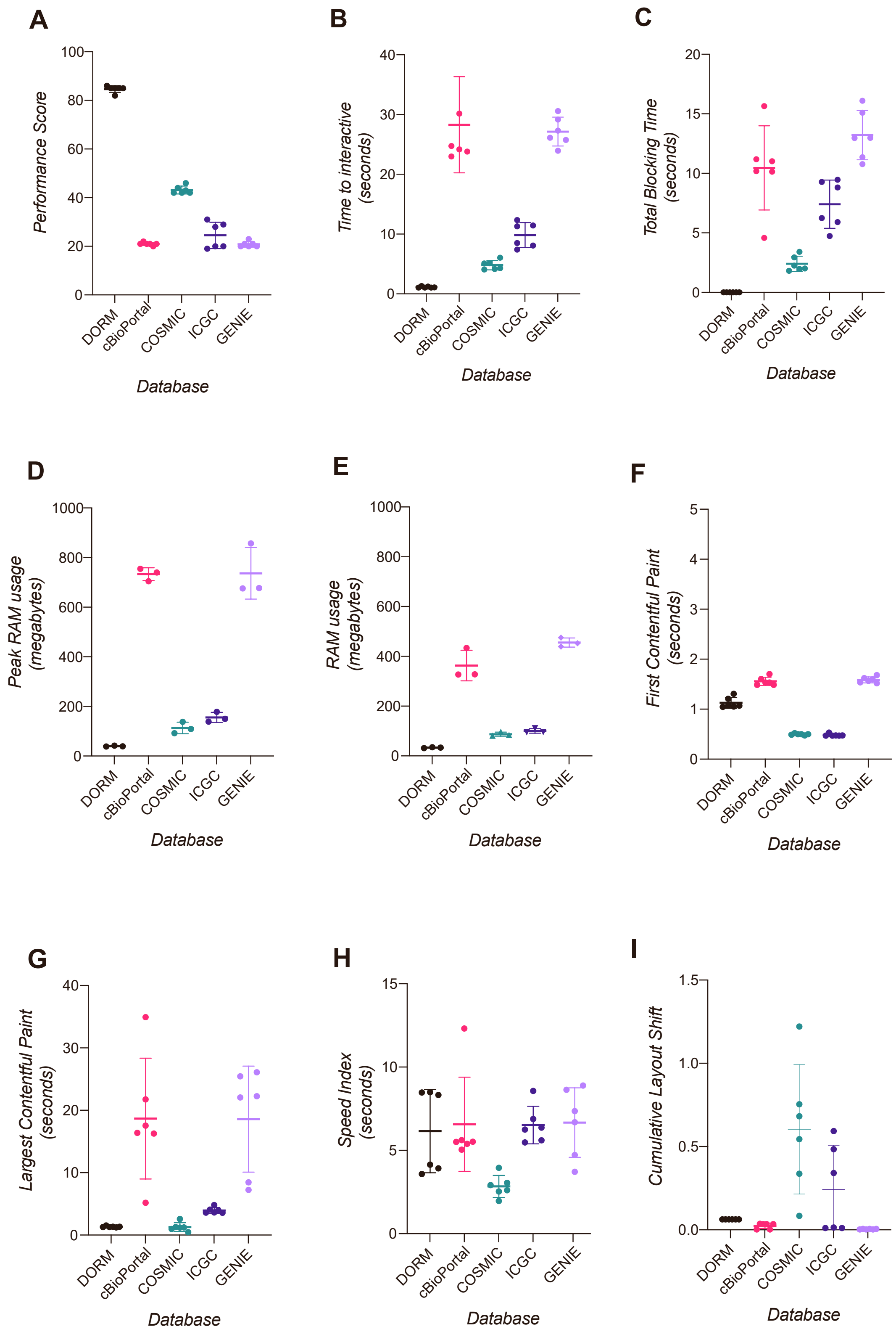

Figure S3. Performance of databases and their websites.
EGFR mutations were searched on each database, and the individual links for that search were used to test the performance of the databases with Google Lighthouse running on Google Chrome web browser. Scatter plots with mean and standard deviation for 3 to six observations for A) Lighthouse performance score, B) Time to interactive (*y*-axis in seconds), C) Total blocking time (*y*-axis in seconds), D) Peak memory (RAM) usage by the web pages (*y*-axis in Megabytes), E) Memory (RAM) usage after garbage collection (browser idling for 2 minutes) while browsing the indicated databases (*y*-axis in Megabytes), F) First contentful paint (*y*-axis in seconds), G) Largest contentful paint (*y*-axis in seconds), H) Speed index (*y*-axis in seconds), I) Cumulative layout shift.

A

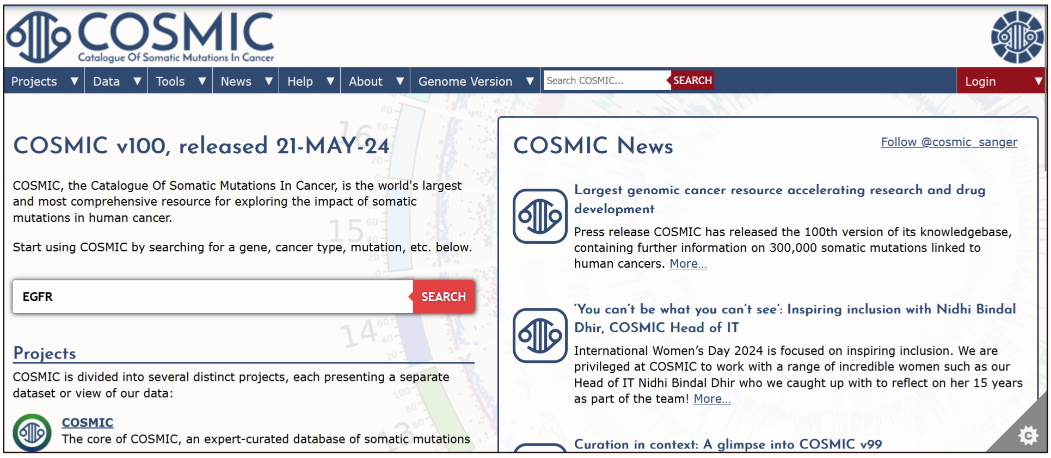

B

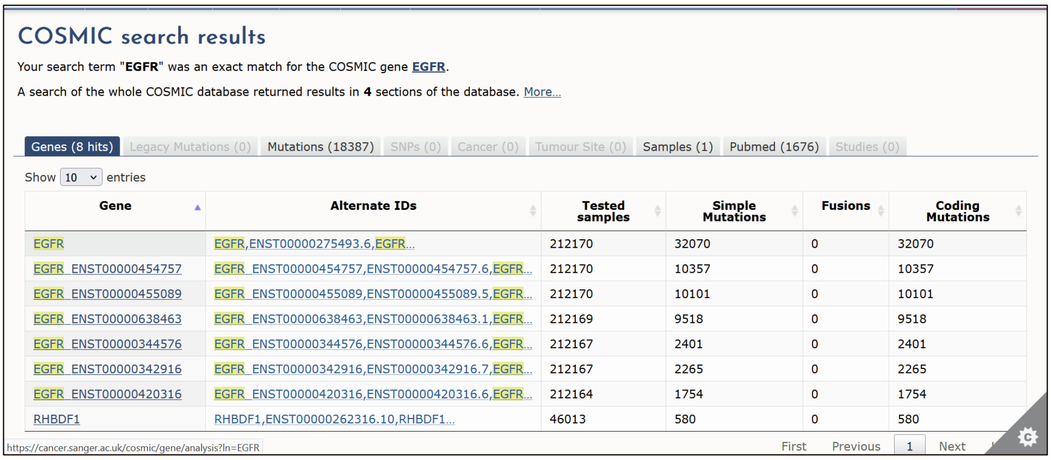

C

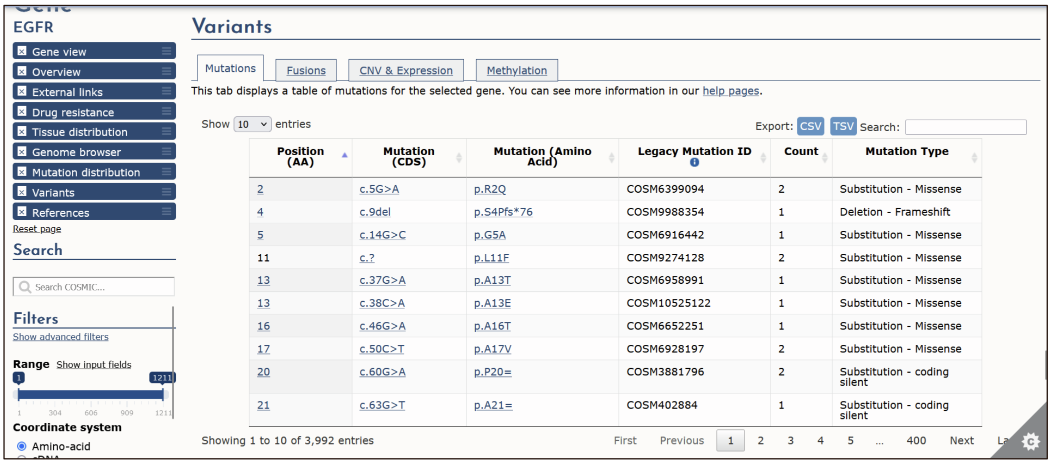

Figure S4. User experience to access mutation frequency information in COSMIC. Screenshots of the user experience to access mutation frequency information through the grpahic user interface of the COSMIC database. A) The query begins by supplying a gene name to the search bar. B) COSMIC requires the user to select the gene/transcript ID to access the mutation frequency information. C) The frequency information can be accessed from the "Variants" table of the Gene page. The frequency information in the table requires further processing since several records for one mutation are listed on the table.

A

B

C

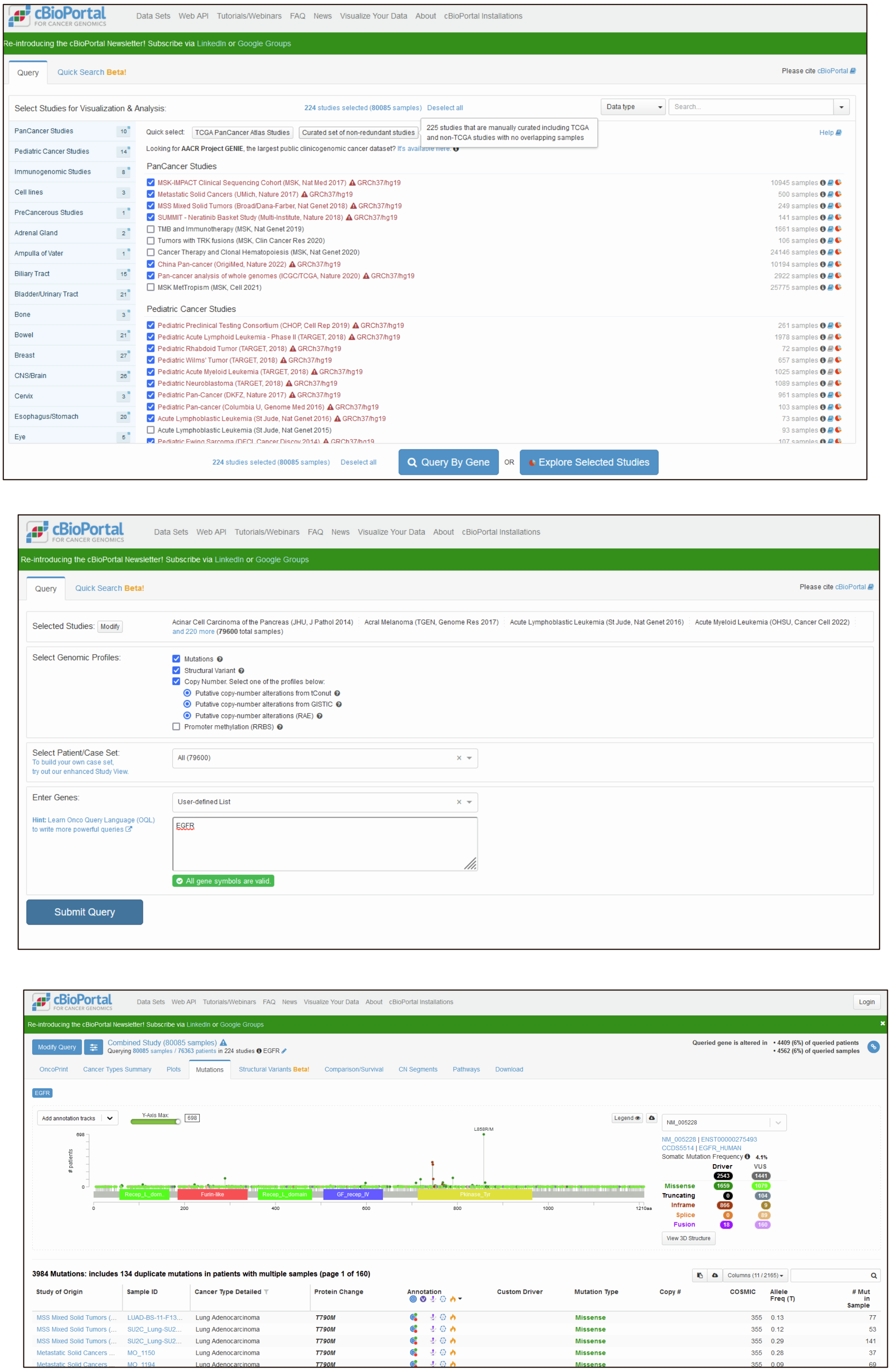

Figure S5. User experience to access mutation frequency information in cBioPortal. Screenshots of the user experience to access mutation frequency information through the graphical user interface of cBioPortal. A) The query begins by selecting studies for the analysis. B) The gene name is supplied to the "Enter Genes"' box that appears after selecting the "Query by Gene" option. C) The frequency information can be accessed from the "Mutations" tab. The frequency information is automatically visualized as a lollipot plot and also as a table. The frequency information in the table requires further processing (summarization) since several sample records for one mutation are listed on the table.

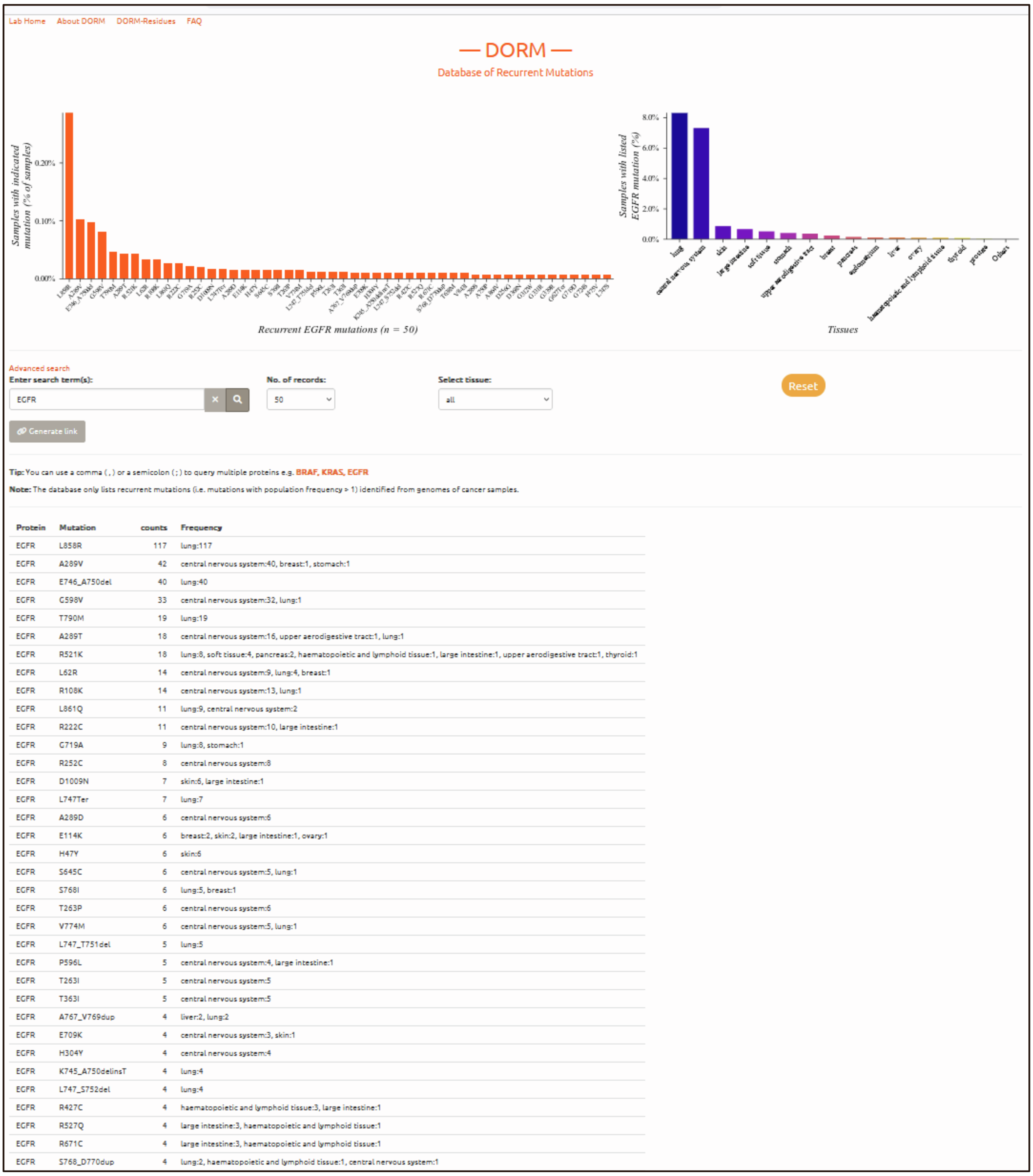

Tip: You can use a comma (,) or a semicolon (;) to query multiple proteins e.g. BRAF, KRAS, EGFR

Note: The database only lists recurrent mutations (i.e. mutations with population frequency > 1) identified from genomes of cancer samples.

Protein	Mutation	counts	Frequency
EGFR	L858R	117	lung:117
EGFR	A289V	42	central nervous system:40, breast:1, stomach:1
EGFR	E746_A750del	40	lung:40
EGFR	G598V	33	central nervous system:32, lung:1
EGFR	T790M	19	lung:19
EGFR	A289T	18	central nervous system:16, upper aerodigestive tract:1, lung:1
EGFR	R521K	18	lung:8, soft tissue:4, pancreas:2, haematopoietic and lymphoid tissue:1, large intestine:1, upper aerodigestive tract:1, thyroid:1
EGFR	L62R	14	central nervous system:9, lung:4, breast:1
EGFR	R108K	14	central nervous system:13, lung:1
EGFR	L861Q	11	lung:9, central nervous system:2
EGFR	R222C	11	central nervous system:10, large intestine:1
EGFR	G719A	9	lung:8, stomach:1
EGFR	R252C	8	central nervous system:8
EGFR	D1009N	7	skin:6, large intestine:1
EGFR	L747Ter	7	lung:7
EGFR	A289D	6	central nervous system:6
EGFR	E114K	6	breast:2, skin:2, large intestine:1, ovary:1
EGFR	H47Y	6	skin:6
EGFR	S645C	6	central nervous system:5, lung:1
EGFR	S768I	6	lung:5, breast:1
EGFR	T263P	6	central nervous system:6
EGFR	V774M	6	central nervous system:5, lung:1
EGFR	L747_T751del	5	lung:5
EGFR	P596L	5	central nervous system:4, large intestine:1
EGFR	T263I	5	central nervous system:5
EGFR	T363I	5	central nervous system:5
EGFR	A767_V769dup	4	liver:2, lung:2
EGFR	E709K	4	central nervous system:3, skin:1
EGFR	H304Y	4	central nervous system:4
EGFR	K745_A750delinsT	4	lung:4
EGFR	L747_S752del	4	lung:4
EGFR	R427C	4	haematopoietic and lymphoid tissue:3, large intestine:1
EGFR	R527Q	4	large intestine:3, haematopoietic and lymphoid tissue:1
EGFR	R671C	4	large intestine:3, haematopoietic and lymphoid tissue:1
EGFR	S768_D770dup	4	lung:2, haematopoietic and lymphoid tissue:1, central nervous system:1

Figure S6. User experience to access mutation frequency information in DORM. Screenshot of the user experience to access mutation frequency information through the user interface of DORM.The gene name is supplied to the search bar and the frequency information is displayed as a bar plot above and as a table below.

Database	Link
DORM	https://eleniuslabtools.utu.fi/tools/DORM/Mutations/?_state_id_=768356cba129db0c
COSMIC	https://cancer.sanger.ac.uk/cosmic/gene/analysis?ln=EGFR
ICGC data portal	https://dcc.icgc.org/genes/ENSG00000146648/mutations
cBioPortal	http://www.cbioportal.org/results/mutations?tab_index=tab_visualize&Action=Submit&session_id=62011bb904dc353874686fc1&plots_horz_selection=%7B%7D&plots_vert_selection=%7B%7D&plots_coloring_selection=%7B%7D
AACR Genie	https://genie.cbioportal.org/results/cancerTypesSummary?cancer_study_list=brca_akt1_genie_2019%2Cglioma_dfci_2020%2Cgenie_public%2Cmbc_genie_2020&Z_SCORE_THRESHOLD=2.0&RPPA_SCORE_THRESHOLD=2.0&profileFilter=mutations&case_set_id=all&gene_list=EGFR&geneset_list=%20&tab_index=tab_visualize&Action=Submit

Table S1. Links to the database searches. The links were used to test the performance of the databases with Google Lighthouse. Results of the performance tests are visualized in Figure S3.

Performing advanced searches on DORM

DORM is a light-weight webtool to browse recurrent mutations in human cancers identified from genome-wide screens (data sourced from COSMIC releases). Here is how to harness the power of regular expressions to craft advanced search queries for the DORM database.

Deconstructing the search

Understanding how the search works *behind-the-scenes* might help you to formulate better and fail-proof queries.

1.The search query is processed line-by-line.

2. A blank space in the query triggers “nesting” of the query parameters from left to right, and is useful when you want to narrow your search space.

Consider the expression:

`KRAS G12 lung|pancreas`

This search expression is processed by extracting all the rows containing the word `KRAS`, then the word `G12` is searched in those results, followed by the regular expression `lung|pancreas`. In practice, this query shows all the KRAS G12 mutations that are reported in samples derived from either the lung or the pancreas.

3. Blank space is **NOT** translated to the OR-operator i.e. `|`, as it is reserved to “nest” search queries (read point #2

above).

4. Comma , and Semicolon ; are provided as delimiters to search sets of proteins. These operators are translated to an OR-operator i.e. `|`. The number of spaces after these symbols do not affect the search i.e. `KRAS,EGFR` and

`KRAS, EGFR` provide the same results.

Using regular expressions

1. **Either - or:**

You can use the `|` operator to for either-or queries, e.g.

KRAS|BRAF - mutations in either the KRAS or BRAF.

EGFR|ERBB - mutations in the proteins EGFR,ERBB2,ERBB3, and ERBB4

N.B. ERBB *matches the ERBB2, 3, 4 and possibly other proteins that start with* ERBB.

2. **Specify inclusions and exclusions:**

You can restrict the exact matches with [] operator. You can specify specific matches, ranges to include or exclude during searches e.g.

Searching NRG gives you several proteins, but if you only want the Neuregulin ligands, you can simply search NRG[1-4] to specifically search for the four Neuregulins. The expression [1-4] is evaluated as the range of numbers [1234].

Additionally, you can exclude results using the [^] operator e.g.

ERBB[^4] - matches all the ERBB2 and ERBB4 among the ERBBs and leaves out ERBB4.

3. **Set word boundaries:**

The word boundary operator \< or \> allows you to fix the boundary of search term. e.g.

\<NRG - excludes proteins which do not begin with NRG

RAS\> - lists all the proteins ending in RAS

RAS\> C\> - lists the mutations in RAS oncogenes that create a change to Cysteine.

Remember, blank space triggers nesting of search operations (point #2 above).

4. **Wildcards:**

Substituting words, numbers etc. is very easy with these:

a. [0-9] or [[:digit:]] - for digits 0-9

b. [a-Z] or [[:alpha:]] - for small alphabets [a-z] and capital [A-Z].

c. The dot . - matches any character

d. [[:space:]] - matches blank space

5. **Match length mofidiers**:

The benefit of using regex is flexibility the wildcard characters offer when you are not sure about the length of matching text. There are 5 main modifiers:

a . * - matches at least 0 times [matching character can be either present or absent]

b. ? - matches exactly 1 time

c. + - matches 1 or more times

d. {n} - matches exactly n times

e. {x,y} - matches at least x, and at most y times. Either x or y can be omitted to create hard upper/lower bounds.

Getting crafty

When you get the hang of regular expressions you can create very specific expressions like this one:

```
ERBB[234]|[HB]{0,1}EGF[R]{0,1}\>|NRG[1-4]|\<EP[GR]\>|AREG|BTC|TGFA
```

This expression searches, all the 4 ERBB-family receptors and the 11 ligands namely: EGFR, ERBB2, ERBB3, ERBB4, AREG, BTC, TGFA, NRG1, NRG2, NRG3, NRG4, EGF, HBEGF, EPG and EGR; and nothing more.

Precision is what you want in science, and regular expressions will get you there :)

Caveats

1. I repeat, Blank space is **NOT** translated to the OR-operator i.e. |, as it is reserved to "nest" search queries (read point #2 above). Please use a comma or semicolon to delimit your list of protein names.

2. The traditional string boundary operators $ and ^ don't work, the reasons are beyond my comprehension. Please use the word boundary operator \>.
